# Home-Based Rehabilitation With Telemonitoring Guidance for Patients With Coronary Artery Disease (Short-Term Results of the TRiCH Study): Randomized Controlled Trial

**DOI:** 10.2196/jmir.9943

**Published:** 2018-06-22

**Authors:** Andrea Avila, Jomme Claes, Kaatje Goetschalckx, Roselien Buys, May Azzawi, Luc Vanhees, Véronique Cornelissen

**Affiliations:** ^1^ Department of Rehabilitation Science KU Leuven Leuven Belgium; ^2^ Department of Cardiovascular Sciences KU Leuven Leuven Belgium; ^3^ Department of Cardiology University Hospital Leuven Leuven Belgium; ^4^ Cardiovascular Research Group School of Healthcare Science Manchester Metropolitan University Manchester United Kingdom

**Keywords:** cardiac rehabilitation, telemonitoring, exercise, coronary artery disease

## Abstract

**Background:**

Cardiac rehabilitation (CR) is an essential part of contemporary coronary heart disease management. However, patients exiting a center-based CR program have difficulty retaining its benefits.

**Objective:**

We aimed to evaluate the added benefit of a home-based CR program with telemonitoring guidance on physical fitness in patients with coronary artery disease (CAD) completing a phase II ambulatory CR program and to compare the effectiveness of this program in a prolonged center-based CR intervention by means of a randomized controlled trial.

**Methods:**

Between February 2014 and August 2016, 90 CAD patients (unblinded, mean age 61.2 years, SD 7.6; 80/90, 89.0% males; mean height 1.73 m, SD 0.7; mean weight 82.9 kg, SD 13; mean body mass index 27.5 kg/m^2^, SD 3.4) who successfully completed a 3-month ambulatory CR program were randomly allocated to one of three groups: home-based (30), center-based (30), or control group (30) on a 1:1:1 basis. Home-based patients received a home-based exercise intervention with telemonitoring guidance consisting of weekly emails or phone calls; center-based patients continued the standard in-hospital CR, and control group patients received the usual care including the advice to remain physically active. All the patients underwent cardiopulmonary exercise testing for assessment of their peak oxygen uptake (VO_2_ P) at baseline and after a 12-week intervention period. Secondary outcomes included physical activity behavior, anthropometric characteristics, traditional cardiovascular risk factors, and quality of life.

**Results:**

Following 12 weeks of intervention, the increase in VO_2_ P was larger in the center-based (*P*=.03) and home-based (*P*=.04) groups than in the control group. In addition, oxygen uptake at the first (*P*-interaction=.03) and second (*P*-interaction=.03) ventilatory thresholds increased significantly more in the home-based group than in the center-based group. No significant changes were observed in the secondary outcomes.

**Conclusions:**

Adding a home-based exercise program with telemonitoring guidance following completion of a phase II ambulatory CR program results in further improvement of physical fitness and is equally as effective as prolonging a center-based CR in patients with CAD.

**Trial Registration:**

ClinicalTrials.gov NCT02047942; https://clinicaltrials.gov/ct2/show/NCT02047942 (Archived by WebCite at http://www.webcitation.org/70CBkSURj)

## Introduction

Cardiovascular diseases (CVD) remain the leading contributor to global premature mortality and morbidity. In Europe, more than 4 million people die from CVD every year, with more than 1.4 million dying before the age of 75 years [1]. Today, secondary prevention of CVD, including coronary artery disease (CAD), by means of cardiac rehabilitation (CR) is considered a class IA recommendation by the European Society of Cardiology, American Heart Association, and American College of Cardiology [2]. CR is now recognized as an essential part of contemporary CAD management that has significantly contributed to the observed reduction in cardiovascular mortality and disability by facilitating the adoption of and adherence to healthy behaviors and promoting an active lifestyle [3]. However, the majority of patients fail to achieve secondary prevention targets in the long term [4]. Many patients receiving center-based CR adopt healthier lifestyles but relapse into old habits when returning to everyday life. After completion of a structured, supervised, exercise-based CR program without any extended support or follow-up, the assumption, of both the participant and CR staff, is that the patient will be able to self-maintain these appropriate health behaviors and optimal CVD risk profile. Unfortunately, studies have shown that patients exiting center-based CR have difficulty retaining the positive benefits derived from their participation [5]. Moreover, previous reports indicate decreased exercise adherence and increased body weight and serum lipid levels as early as 6 months after CR [4,6].

Consequently, there is a need for innovative CR methods to increase long-term adherence to a physically active lifestyle that will result in more sustained effects on health-related physical fitness and cardiovascular health, thus, reducing morbidity and mortality [7]. One attractive strategy is the use of home-based exercise training in combination with telemonitoring guidance. Home-based programs may overcome barriers associated with participation in a center-based exercise program, and they have been shown to provide comparable long-term effects on mortality, recurrent coronary event risk, and cardiovascular risk factors in patients with CVD [8]. This has been attributed partly to the fact that home-based interventions focus more on the development of self-regulatory techniques that create empowerment and perceived control, resulting in longer lasting effects on physical activity improvements [9]. That is, individuals who develop their own physical activity plans are more likely to adhere to these plans than those who have a structured exercise plan imposed on them [9]. The use of information and communication technology to augment home-based programs also enables the provision of additional feedback, education, and counseling [8].

A recent meta-analysis by Buckingham et al [10] found no significant differences in the short-term (<12 months) or long-term (>12 months) patient outcomes including exercise capacity, modifiable risk factors (blood pressure, blood lipid concentrations, and smoking), health-related quality of life (HRQoL), and cardiac events (mortality, coronary revascularization, and hospital readmissions) among patients participating in home-based or center-based phase II CR. However, there is little evidence about the added benefits of a home-based exercise program for patients being discharged from center-based CR compared with advice only.

In this paper, we report on the secondary objective of the TeleRehabilitation in Coronary Heart disease (TRiCH) study. We aimed to investigate the short-term effect of an HR CR program with telemonitoring guidance on physical fitness and other secondary outcomes in CHD patients following completion of a center-based CR program. We also aimed to compare the effectiveness of this program with that of a prolonged center-based CR program by means of a randomized controlled trial (NCT02047942). The longer-term results of the TRiCH study will be published in a second report.

## Methods

### Study Design

We conducted a randomized controlled trial using a three-arm, parallel group design among 90 low-to-moderate risk CAD patients completing a phase II CR program at the University Hospital Leuven (Belgium). The study protocol was approved by the medical ethical committee of the UZ Leuven/KU Leuven. The protocol has been described in detail elsewhere [7].

### Patient Population and Randomization

Patients were recruited between February 2014 and August 2016 at the University Hospital Leuven (Belgium). The eligible patients included men and women (aged between 40 and 75 years) with angiographically-documented CAD or previous myocardial infarction, on optimal medical treatment for the last 6 weeks, who successfully completed a supervised ambulatory CR program and who had access to a computer with Internet connection. The exclusion criteria included known clinically significant ventricular arrhythmia or exercise-induced arrhythmia at screening, myocardial ischemia, other cardiac diseases (valve disease with significant hemodynamic consequences, hypertrophic cardiomyopathy, etc), significant illness for the last 6 weeks, co-morbidity that might represent a significant influence on 1-year prognosis (eg, cancer), and co-morbidity that limits exercise testing and/or training. The criteria for ischemia on the electrocardiogram during exercise included horizontal or downsloping ST depression ≥1 mm at 80 ms after the J-point or any ST depression >1 mm at 80 ms after the J-point [11]. The eligible patients were contacted in the last weeks of their in-hospital ambulatory CR program (phase II) and were provided verbal information about the TRiCH study. Agreeing patients subsequently received written information and were asked to provide written informed consent according to the principles of Good Clinical Practice and the Declaration of Helsinki.

### Procedures

All the agreeing patients who had completed 40 sessions of their ambulatory CR program (phase II) were included and were subsequently randomized in a 1:1:1 ratio to one of three groups: home-based group, center-based group, or a usual care control group by means of a web-based random number generator.

The home-based CR group received training for the first three sessions under the supervision of the investigator. During this period, the patients received an individualized aerobic exercise prescription recommending at least 150 min of exercise per week (preferably 6-7 days/week) at an individually determined target heart rate corresponding to a moderate intensity (ie, 70%-80% of heart rate reserve [HRR]) in their home environment during the 12-week intervention. Furthermore, this group received instructions on how to use the heart rate monitor (Garmin Forerunner 210, Wichita USA) and how to upload their exercise data to the Garmin platform [12]. This application was used to review the training data by both the patient and the investigator [13]. Patients received feedback via phone or email once a week according to their preferences. These contact moments were used for the following purposes: 1) to check for adverse events and injuries, 2) to provide feedback on performed exercise during the preceding week, 3) to discuss the exercise program regarding duration and intensity, and 4) to discuss adherence and barriers to adherence if necessary.

Patients randomized to center-based CR continued their exercise program at the outpatient clinic of UZ Leuven under the direct supervision of physiotherapists. The patients were asked to perform three exercise sessions per week totaling approximately 150 min of endurance exercise. Each training session consisted of predominantly endurance training (2×7 min of cycling, 2×7 min of treadmill walking/running, 7 min of arm ergometry or rowing, and 2×7 min of dynamic calisthenics) and was followed by relaxation. The endurance exercise workload was individually controlled by heart rate monitoring, which was performed by palpation by the physiotherapist during the last minute of each round of exercise. Exercise load was adjusted to maintain target heart rate (70%-80% of the HRR). Patients randomized to the control group received usual care including the standard advice to remain physically active.

### Primary Outcome Measure

Primary outcome was change in the exercise capacity following the intervention. Exercise capacity (defined as the maximum amount of physical exertion that a patient could sustain) [14] was determined at baseline and at the end of the intervention using a maximally graded test on a bicycle with breath-by-breath respiratory gas analysis (Ergometrics 800S, Ergometrics, Bitz, Baden-Württemberg, Germany). Peak exercise capacity was defined as the 30-s average oxygen uptake (VO_2_) at the highest workload [7]. Ventilatory thresholds (VTs), peak respiratory exchange ratio, and peak heart rate were also established [7].

### Secondary Outcome Measures

Secondary outcomes included daily physical activity, measured using a Sensewear Mini Armband (BodyMedia, Inc., Pittsburgh, PA, USA). Steps, sedentary time (duration of sedentary activity at an intensity of ≤1.5 metabolic equivalents of task [METs], min), active energy expenditure (physical activity at an intensity of ≥3 METs, kcal), and duration of moderate and vigorous physical activity (≥3 METs, min) were used in the analyses [15]. Oxygen uptake on-kinetics were established at least 48 h after the maximal exercise test and was calculated algebraically and expressed as mean response time [7]. Sitting-rising test (SRT), handgrip strength (JAMAR grip strength dynamometer), and quadriceps maximal isometric knee extension strength and endurance (Biodex Medical Systems Inc., 840-000 System 4, New York, USA) were obtained along with traditional cardiovascular risk factors such as anthropometric measures (body mass index, waist and hip circumference) and biochemical parameters of a fasting blood sample (glucose, total cholesterol, low-density lipoprotein [LDL] cholesterol, high-density lipoprotein [HDL] cholesterol, and triglycerides). Additionally, homeostasis assessment model (HOMA) [16] index was calculated using the following formula: fasting plasma glucose (mmol/L) times fasting serum insulin (mU/L) divided by 22.5. Low HOMA-IR values indicated high insulin sensitivity, whereas high HOMA-IR values indicated low insulin sensitivity (insulin resistance). For this study, patients with HOMA-IR ≥ 3.8 were considered to be insulin resistant [17]. Finally, HRQoL was obtained by means of the standard version of the Short Form 36 [7].

### Analysis

All data were expressed as mean (SD) or median, range, or percentages (for categorical variables). Statistical analyses were performed using SPSS (version 20; SPSS for windows; SPSS Inc., Chicago, IL). Shapiro–Wilk test was used to assess normality. At baseline, the groups were compared using one-way analysis of variance or chi-square tests. For follow-up data, a linear mixed modeling method was used to evaluate time, group, and time × group interaction effects. The analysis was complemented with a matrix syntax code including a least significant difference post-hoc test when a significant time × group interaction identified a group that significantly differed over time. An intention-to-treat analysis was performed on the primary outcome (peak oxygen uptake, VO_2_ P), and on-treatment analysis was used for secondary outcomes. Spearman correlation coefficients (*p*) were calculated between VO_2_ P and active energy expenditure and physical activity duration at 12 weeks. A probability level of *P* ≤ 0.05 was considered significant.

## Results

**Figure 1:**
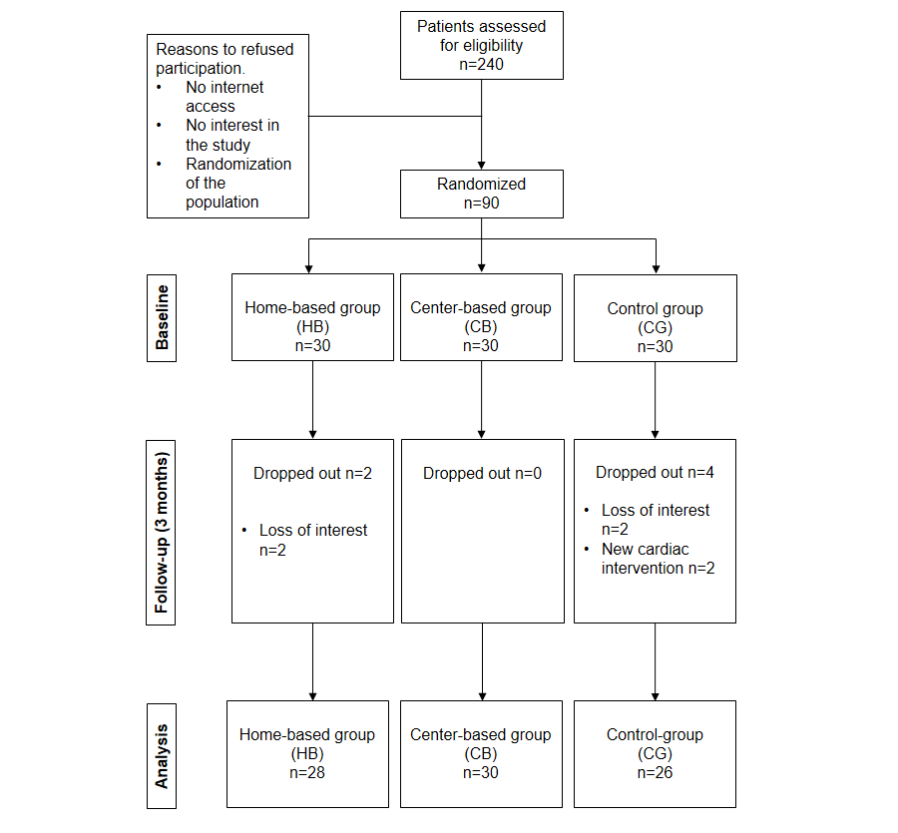
Flow of patients through the study.

A total of 90 CAD patients agreed to participate and were randomized to home-based group (n=30), center-based group (n=30), and control group group (n=30). Figure 1 shows the flow of patients throughout the study. Six patients, (4 men: control group, n=4; home-based, n=2) dropped out during the 3-month intervention period. Reasons for dropout included loss of interest (control group, n=2; home-based, n=2) and a new cardiac intervention (ie, percutaneous coronary intervention) (control group, n=2). No serious adverse events related to exercise occurred in any of the groups.

The basic characteristics of the study population are described in Table 1. The mean age of the participants was 61.4 (SD 7.3) years (range: 42-73 years). A total of 10 (11.1%) women participated in the study, and patients were on average slightly overweight, 27.5 (SD 3.4 kg/m^2^). Overall, exercise capacity was normal, 101.1% (SD 21.1) compared with reference values [18]. Baseline characteristics were comparable between the groups regarding physical characteristics, reason for referral, and pharmacological therapy.

### Primary Outcome Measure

Changes in cardiorespiratory parameters are described in Table 2. As can be appreciated from peak respiratory exchange ratios (RER), participants in all three groups exerted a similar maximal effort at baseline and follow-up. The pattern of change in VO_2_ P differed significantly over time among the three groups (group × time interaction, *P*=.04), with a larger improvement following home-based (*P*=.03) and center-based (*P*=.04) interventions than control group interventions. Group × time interactions were also established for O_2_ uptake at the first ventilatory threshold (VT_1_; *P*-interaction=.03) and the second ventilatory threshold (VT_2_; *P*-interaction=.03), with larger improvements in the home-based group than in the control group.

### Secondary Outcome Measures

Changes in daily physical activity are shown in Table 3. Physical activity behavior remained constant after the intervention (*P*-time=.73). Of all the patients, 97.0% (84/90) met the international guidelines of 150 min or more of moderate physical activity per week [19]. In addition, a significant increase in sedentary time in the center-based group (*P*-interaction=.02) was found. No significant correlation of change in VO_2_ P with change in active energy expenditure (Spearman p=−.14; *P*=.40) or change in physical activity duration (ρ=.09; *P*=.44) was found. However, a significant correlation of VO_2_ P with physical activity duration (ρ= .53; *P*<.001) at 12 weeks as well as with active energy expenditure (ρ=.37; *P*<.001) was found.

**Table 1:** Baseline characteristics of patients.

Characteristics			Home-based (n=30)	Center-based (n=30)	Control (n=30)
**General characteristics**					
	Age (years), mean (SD)		58.6 (13)	61.9 (7.3)	61.7 (7.7)
	Female, n (%)		4 (13)	3 (10)	3 (10)
	% of Predicted peakVO_2_^a^, mean (SD)		99.9 (23.1)	99.3 (20.1)	105.2 (20.2)
**Reason for referral, n (%)**					
	CABG^b^		18 (60)	18 (60)	20 (67)
	PCI^c^		12 (40)	12 (40)	10 (33)
**Cardiovascular risk factors, n (%)**					
	Familial predisposition		12 (40)	8 (27)	13 (43)
	Hypertension		14 (47)	11 (37)	17 (57)
	Diabetes		12 (40)	8 (27)	4 (13)
	Dyslipidemia		15 (50)	17 (57)	19 (63)
	**Smoking**				
		Never-smoker	11 (37)	14 (47)	15 (50)
		Ex-smoker	16 (53)	15 (50)	15 (50)
		Current-smoker	3 (10)	1 (3)	0 (0)
**Medication, n (%)**					
	Anti-hypertensive^d^		23 (77)	27 (90)	24 (80)
	Beta Blockers		21 (70)	23 (77)	25 (83)
	Statins		28 (93)	29 (97)	28 (93)
	Aspirin		29 (97)	27 (90)	29 (97)
	Anti-thrombotic		19 (63)	18 (60)	23 (77)
	Anti-arrhythmic		1 (3)	1 (3)	0 (0)
	Hypoglycemic		4 (13)	8 (27)	4 (13)
	Vasodilators		0 (0)	1 (3)	2 (7)

^a^VO_2_: oxygen uptake.

^b^CABG: coronary artery bypass graft**.**

^c^PCI: percutaneous coronary intervention.

^d^Anti-hypertensive medication: warfarine and clopidogrel.

**Table 2:** Changes in cardiorespiratory parameters at baseline and 3-month follow-up.

Parameter	Home-based (n=28), mean (SD)		Center-based (n=30), mean (SD)		Control (n=26), mean (SD)		*P* value		
	Baseline	3-Month	Baseline	3-Month^a^	Baseline	3-Month	Time	Group	Interaction
VO_2_ peak (mL•kg^−1^•min^−1^)	26.7 (6.55)	27.8 (6.83)	25.4 (7.32)	26.7 (7.90)	26.6 (4.97)	26.4 (5.42)	.08	.69	.04^,b^
VT1^a^ (mL•kg^−1^•min^−1^)	19.5 (1.07)	21.5 (1.07)	19.5 (1.04)	20.4 (1.04)	19.9 (1.08)	19.3 (1.11)	.81	.06	.03^,b^
VT2^c^ (mL•kg^−1^•min^−1^)	24.9 (5.25)	26.3 (6.98)	22.7 (6.95)	24.2 (7.13)	24.7 (5.08)	22.9 (4.19)	.41	.49	.03^,b^
Duration (s)	570 (136)	587 (157)	549 (133)	552 (157)	600 (126)	579 (116)	.89	.52	.23
Peak heart rate (bpm)	140 (18.8)	139 (17.8)	141 (21.5)	140 (21.1)	140 (18.9)	140 (16.6)	.75	.97	.95
Peak load (watts)	198 (49)	200 (54)	191 (50)	191 (54)	206 (41)	197 (38)	.39	.67	.15
Peak RER^d^	1.24 (0.89)	1.21 (0.10)	1.23 (0.80)	1.24 (0.10)	1.20 (0.8)	1.20 (0.13)	.47	.32	.36
Borg scale	15.8 (1.16)	15.8 (1.33)	16.2 (1.04)	16 (1.17)	15.9 (1.05)	16.2 (1.02)	.87	.45	.38

^a^VT1: first ventilatory threshold.

^b^*P-* interaction<.05.

^c^VT2: second ventilatory threshold.

^d^RER: respiratory exchange ratios.

**Table 3:** Changes in daily physical activity at baseline and 3-month follow-up.

Physical activity	Home-based (n=24), mean (range)		Center-based (n=28), mean (range)		Control (n=26), mean (range)		*P* value		
	Baseline	3-Month	Baseline	3-Month	Baseline	3-Month	Time	Group	Interaction
Steps per day	7896 (2018-12554)	6469 (473-12828)	7608 (2474-13281)	7065 (489-14785)	6419 (2227-13181)	6408 (296-12041)	.18	.56	.18
Sedentary time (≤1.5 METs; min/day)	1039 (688-1260)	1032 (790-1455)	1005 (122-1290)	1094 (857-1254)^a^	1100 (825-1355)	1062 (484-1402)	.56	.43	.02^b^
Active energy expenditure (>3METs; kcal)	1336 (351-3217)	1307 (661-2246)	1137 (484-2539)	1244 (549-2745)	1223 (401-2253)	1196 (181-2510)	.56	.40	.45
Physical activity duration (>3METs; min/day)	145 (34-299)	141(51-259)	146 (28-417)	134 (29-366)	114 (30-311)	114 (6-382)	.73	.27	.47
Moderate physical activity duration (3-6 METs; min/day)	136 (34-238)	134 (49-241)	140 (28-391)	128 (27-348)	109 (29-303)	115 (25-368)	.57	.24	.62
Vigorous physical activity duration (>6 METs; min/day)	8 (0-33)	7 (0-24)	6 (0-26)	6 (0-24)	5 (0-20)	2 (0-27)	.50	.21	.59

^a^METs: metabolic equivalents of task.

^b^*P-* interaction <.05.

**Figure 2:**
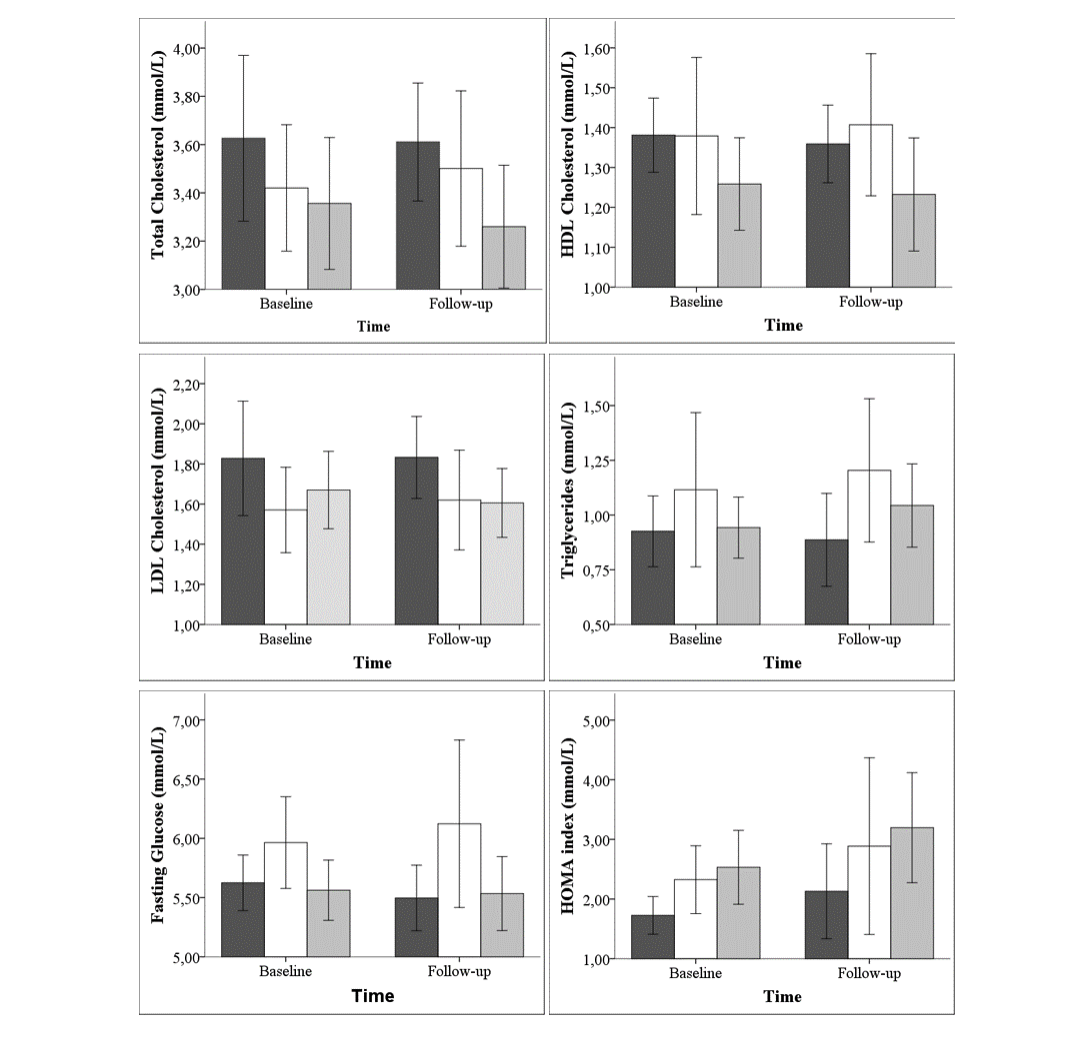
Cardiovascular risk factors.No significant changes were found in cardiovascular risk factors for total cholesterol (*P*-interaction=.82), HDL-cholesterol (*P*-interaction=.69), LDL-cholesterol (*P*-interaction=.79), triglycerides (*P*-interaction=.27), fasting glucose (*P*-interaction=.71), HOMA index (*P*-interaction=.93). Dark gray column: home-based group, White column: center-based group, Light grey column: control group. HDL: high-density lipoprotein; HOMA: homeostasis assessment; LDL: how-density lipoprotein.

As shown in table Table 4, isometric handgrip strength (HG), isometric quadriceps strength, and endurance, as well as exercise-onset oxygen uptake on-kinetics remained stable during the follow-up period. Additionally, cardiovascular risk factors (Figure 2) and anthropometrics (Table 5) were similar between the groups at baseline and remained stable during the follow-up period, except for an increase in HOMA index (*P*-time=.05), which was not different between the groups. Finally, there were no significant changes in the overall score for HRQoL (*P*-interaction=.57) as well as the physical (*P*-interaction=.50) and mental (*P*-interaction=.85) composite scores. Table 6 shows HRQoL from baseline and follow-up evaluations.

### Training Data

Patients in the home-based group completed an average of 2.5 sessions per week (range: 12-60 sessions for 12 weeks), whereas those in the center-based group completed an average of 2.0 sessions per week (range: 4-36 sessions for 12 weeks). Patients in the home-based group exercised for an average 164 min per week at an average intensity of 46.8% of HRR (76.7 min within the prescribed zone). Patients in the center-based group exercised for an average 90 min per week at an average intensity of 61.2% of HRR.

**Table 4:** Changes in muscle strength and exercise-onset oxygen uptake (VO_2_) kinetics.

Parameter		Home-based (n=23), mean (SD)		Center-based (n=29), mean (SD)		Control (n=19), mean (SD)		*P* value		
		Baseline	3-Month	Baseline	3-Month	Baseline	3-Month	Time	Group	Interaction
**Muscle strength**										
	Handgrip strength (kg)	43.1 (10.5)	44.7 (12.3)	40.2 (8.6)	41.2 (8.3)	41.6 (8.3)	43.8 (9.3)	.99	.23	.23
	Isometric quadriceps extension (60° Nm)	151.8 (28)	164.1 (37)	150.5 (44.9)	155 (43.4)	148.7 (30)	148.8 (28.3)	.23	.47	.86
	Extension total work (J)	1614 (680)	1976 (718)	1758 (756)	1893 (717)	1694 (796)	1906 (689)	.09	.94	.52
**Exercise-Onset VO**_2_^i^ **kinetics**										
	Average MRT^a^ (s)	45.5 (16.2)	39.8 (9.3)	38.7 (8.1)	40.8 (9.1)	39.8 (16.9)	43.6 (22)	.98	.64	.19

^a^MRT: mean response time.

**Table 5:** Outcomes at baseline and 3-month follow-up regarding anthropometric parameters.

Anthropometrics	Home-based (n=28), mean (SD)		Center-based (n=30), mean (SD)			Control (n=26), mean (SD)			*P* value				
	Baseline	3-Month		Baseline	3-Month		Baseline	3-Month		Time		Group	Interaction
Weight (kg)	80.4 (10.3)	79.8 (10.3)		82.9 (15.3)	82.4 (15)		85 (12.7)	84.1 (11.9)		.36	.22		.55
Body mass index (kg/m^2^)	26.6 (2.5)	26.4 (2.5)		27.8 (4)	27.6 (4.1)		28 (3.3)	27.6 (2.6)		.28	.21		.53
Body fat (%)	26.8 (5.7)	26.5 (6.1)		29.5 (5.5)	28.4 (6.4)		29.5 (5.1)	28.8 (4.2)		.16	.12		.60
Waist Circumference (cm)	96.8 (8.8)	95.2 (8.2)		98.7 (11.2)	99 (11.4)		99.5 (9.8)	99.6 (9.6)		.81	.29		.11
Hip circumference (cm)	101.1 (5.9)	101 (5.4)		102.6 (7.4)	102.7 (7.6)		102.5 (4.1)	102.1 (5.1)		.31	.42		.82
Systolic blood pressure (mmHg)	125.3 (15.6)	124.1 (13.9)		127.4 (15)	124.1 (13.8)		123.6 (13)	122.9 (14)		.29	.75		.73
Diastolic blood pressure (mmHg)	75.4 (9.5)	75.8 (9)		76 (8.3)	74.7 (8.2)		75.5 (8.9)	76.9 (7.9)		.81	.91		.37
Heart rate at rest (bpm)	56.8 (9.1)	56.4 (7.4)		57.1 (8.2)	57.7 (10.1)		56 (8.1)	56.7 (14)		.90	.68		.84

**Table 6:** Changes in health-related quality of life (HRQoL).

HRQoL measures	Home-based (n=28), mean (SD)		Center-based (n=30), mean (SD)		Control (n=26), mean (SD)		*P* value		
	Baseline	3-Month	Baseline	3-Month	Baseline	3-Month	Time	Group	Interaction
Physical Function	90 (10.7)	91.9 (7.6)	85.3 (15.6)	87.4 (13.6)	81.4 (15.7)	84.3 (16.1)	.19	.04	.49
Role-physical function	83.6 (27.3)	87.5 (28.4)	74.2 (36.6)	83.9 (31.3)	58 (41.4)	61.4 (38.9)	.15	.00	.43
Bodily pain	84.2 (13.7)	80.9 (17.7)	82.5 (19.2)	83.8 (23.3)	72.7 (23.3)	77.6 (23.2)	.83	.11	.70
General health	74.2 (13.1)	75.2 (18.7)	75.7 (13.6)	72.4 (22.9)	64.2 (16.6)	71.5 (19.1)	.41	.17	.17
Vitality	72.8 (13.9)	71 (13.4)	69.3 (15.6)	74.6 (15.4)	65.3 (14.6)	63.9 (20)	.70	.06	.10
Social function	87.6 (16.7)	90.6 (16.5)	86.6 (17.3)	89.1 (15.7)	83.7 (18.2)	89.5 (17.5)	.20	.81	.90
Role-emotional function	84.6 (27)	86.8 (22.8)	84.5 (33.3)	86.8 (29.1)	86.2 (30.2)	84.7 (31)	.73	.99	.79
Mental health	78.6 (15.6)	77.5 (17.6)	79.3 (13.3)	81.4 (15)	78.2 (15.6)	76.6 (21.1)	.91	.72	.59
Physical Composite Score	80.9 (12.3)	81.3 (13.1)	77.2 (15.2)	80.8 (16.3)	67.9 (17.1)	71.8 (18.3)	.008	.07	.50
Mental Composite Score	79.8 (13.7)	80.2 (13.5)	77.5 (18)	81.3 (15.5)	75.1 (13.8)	77.2 (16.2)	.06	.38	.85
SF-36^a^	82.2 (13.3)	82.6 (13)	79.8 (16.1)	82.6 (15.8)	73.3 (15.1)	76.4 (16.4)	.07	.06	.57

^a^SF-36: Short Form 36. Scores of the domains of the SF-36: 0=worst and 100=best score.

## Discussion

The significant finding of our randomized controlled study is that a 3-month home-based rehabilitative intervention with telemonitoring guidance results in further improvement of exercise capacity (VO_2_ P) in CAD patients who had recently completed a phase II ambulatory program, and a home-based program is as effective as a prolonged center-based CR. The observed improvements of 1.30 mL·kg^−1^·min^−1^and 1.10 mL·kg^−1^·min^−1^in VO_2_ P of center-based and home-based groups, respectively, are likely to be clinically relevant. It has been shown in earlier studies that a 1 mL·kg^−1^·min^−1^increase in exercise capacity is associated with a 10% reduction in cardiovascular mortality [20]. Thus, our results support the added value of a structured continued rehabilitation program.

There are only few studies in the literature that have investigated the effectiveness of a home-based, telemonitored, phase III CR program starting immediately after completion of a phase II center-based CR program. A small proof-of-concept study by Brubaker et al [5] randomly assigned 31 patients to home-based, center-based, or standard care. In line with our results, they found that the home-based program was as effective as the center-based program at improving and maintaining oxygen consumption among patients 9 months after exiting a CR program. In the Telerehab III trial [21], 140 patients were randomized to a telerehabilitation program in addition to conventional CR or conventional CR alone. This study also reported that a 6-month patient-specific comprehensive telerehabilitation program initiated 6 weeks after the start of ambulatory rehabilitation leads to a bigger improvement in VO_2_ P and confirmed our results of lack of an additional weight loss, blood pressure reduction, lipid profile improvement, and glycemic control.

In the last decade, several meta-analyses have been published demonstrating the effectiveness of home-based programs for CAD patients implemented as a phase II CR program [22-24]. Although the increase in VO_2_ P of 4%-5% in our phase III center-based and home-based groups is less than what has been seen in previous phase II programs, this is still of clinical relevance, as the purpose of phase III (maintenance phase) CR is to preserve, or if possible, enhance the health benefits gained in phase II. Our results further show that although patients in the home-based group exercised only 75 min per week at the prescribed intensity and the average intensity was below the recommended thresholds, patients were still able to further increase their exercise capacity compared with those in the control group who receive only advice on how to maintain a physically active lifestyle. This is in line with meta-analytic results of Swain and Franklin [25], demonstrating that in healthy individuals with a mean baseline VO_2max_<40 mL·kg^−1^·min^−1^, there is no clear minimal intensity threshold to increase their aerobic capacity and that patients already show improvements when exercising at an intensity of 40% of HRR. Yet, there is abundant evidence that larger effects on health and fitness are established when individuals exercise at higher intensities and larger duration [26].

Regarding physical activity, Ayabe et al [27], reported that 6500-8500 steps/day should be considered as the minimal and optimal goal of physical activity for secondary prevention of CVD. With the number of steps ranging between 7612 (home-based) and 7700 (center-based), the patients in the home-based and center-based groups were within this target zone, whereas those in the control group (5566) did not seem to reach this goal. However, we were not able to promote an additional increase in the number of daily steps. These results are in line with previous studies that have demonstrated how exercise interventions focused on physical fitness improvement in cardiac patients do not influence the improvement of physical activity [21,28,29].

We observed, however, a small but significant increase in sedentary time in the center-based group. Evidence suggests that those participating in exercise-focused interventions are not likely to reduce their sedentary time by a meaningful amount [30]. King et al [31] explained this behavior as a compensatory effect for exercise. That is, the simple fact of enrolling in a supervised exercise program might reduce physical activity levels throughout the rest of the day. This compensatory effect acts in different ways by promoting adjustments in energy expenditure in order to save energy or recover from the exercise training, consequently, increasing the sedentary time [32]. Growing evidence suggest that prolonged sedentary behavior can affect cardiovascular and all-cause mortality risk independent of physical activity [33]. Probably, recommendations on sedentary behavior can be included in CR programs, given the current state of the evidence [30].

Small improvements in weight reduction and body composition as well as in blood pressure were observed post-intervention, although this did not reach significance. This is consistent with the findings in the Telerehab III trial [21]. According to Frederix et al, digital health interventions seem to be able to improve cardiovascular risk factors in primary prevention, but not in secondary prevention, programs [21]. Furthermore, we hypothesize that when the pharmacological management of the groups is close to optimal, like in our patients, the incremental benefit of secondary prevention programs over usual care is very small [34].

One of the main objectives of CR is to optimize patients’ physical functionality as a means to improve quality of life. In our study, no effects of our interventions on different HRQoL domains could be found. Contrary to our results, Smith et al [35] found clinically significant improvements in HRQoL in their home-based group compared with their hospital-based group after a 6-month intervention. The authors considered that 6 months of CR, regardless of location, was associated with improvements in physical HRQoL. Thus, it is possible that with a longer intervention for both the groups, some differences could have been obtained in our study.

One area that is not commonly considered with CR is the functional status or abilities of the patient. The typical patient in CR is over the age of 60 and presents with multiple cardiovascular risk factors, such as inactivity and obesity, and is recovering from a recent cardiac event. All these factors can lead to deficits in balance, mobility, and function. Sumide et al, found that musculoskeletal fitness and flexibility, measured by SRT, was a significant predictor of mortality in 51- to 80-year-old participants [36]. In this study, no significant difference was seen in SRT between the groups (*P*=.36). Our results are in line with those of Oerkild et al [37]. Regarding components of muscular fitness, Mroszcwyk et al [38] and Thomaes et al [39] found increased HG strength in patients following 3 months of a (predominantly aerobic exercise training) CR program. In our study, however, no changes were observed after 12 weeks of intervention. It is possible that the largest gains in HG strength appear during phase II of CR, while our intervention targeted phase III patients. In addition, we found no significant difference in knee extension strength or resistance after 3 months of home-based or center-based training, which may be explained by the focus on aerobic training during the intervention. These results are also consistent with those of previous studies [40,41]. Conraads et al collected muscle strength data in 75 CAD patients before and after 12 weeks of an aerobic interval training or continuous training CR program, finding that muscle strength did not improve. The authors considered as a possible cause the use of statins that has been associated with negative side effects on the muscles. In our study, 94% (79/84) of the patients are treated with statins, although insufficient evidence exists to prove that statins really affect muscle strength [42]. Currently, there is very limited evidence regarding the effects of telerehabilitation on muscle strength, and more research is needed [43].

Finally, while short-term changes are of interest, it is important to establish whether the benefits are maintained over time; thus, further research should focus on the long-term effects of home-based CR with telemonitoring guidance.

### Limitations

Our study should be interpreted within the context of its limitations. First, next to physical activity training, CR includes other important core components such as nutritional counseling, risk factor management, and psychosocial management. Although physical activity training comprises 30%-50% (up to >70%) of all CR activities, it should be acknowledged that this study evaluates the effect of physical activity telemonitoring rather than telerehabilitation [3]. Second, heart rate monitors were used only in the home-based group as we opted to not change the traditional center-based program where heart rate is measured by palpation by physiotherapists. We were not able to precisely define the exact number of minutes patients spend within the prescribed training zone.

Another limitation of this study is the lack of blinding of test personnel. However, as the main outcome measure was VO_2_ P, the effort of the participants can be objectively quantified by means of RER and subjectively by means of the Borg scale [44]. The study, as in most randomized controlled trials, has missing outcome data. Regarding muscle strength, 19 values were completely missing at random due to technical problems, and regarding physical activity, the data was incomplete and, thus, excluded for 12 patients.

### Conclusion

The results of our study show that home-based CR with telemonitoring guidance can be an effective alternative to center-based CR for further improving exercise capacity following phase II CR in CHD patients.

## Appendix

Multimedia Appendix 1 CONSORT‐EHEALTH checklist (V 1.6.1).

## Abbreviations

CAD: coronary artery disease
CHD: coronary heart disease
CR: cardiac rehabilitation
CVD: cardiovascular diseases
HDL: high-density lipoprotein
HG: hand grip
HOMA: homeostasis assessment
HRQoL: health-related quality of life
HRR: heart rate reserve
LDL: how-density lipoprotein
RER: hespiratory exchange ratios
SRT: sitting-rising test
TRiCH: TeleRehabilitation in Coronary Heart
VO_2_P: peak oxygen uptake
VT: ventilatory threshold
